# Inherited duplication of the pseudoautosomal region Xq28 in a subject with Gilles de la Tourette syndrome and intellectual disability: a case report

**DOI:** 10.1186/s13039-020-00493-3

**Published:** 2020-06-22

**Authors:** Stefania Maccarini, Annamaria Cipani, Valeria Bertini, Jelena Skripac, Alessandro Salvi, Giuseppe Borsani, Eleonora Marchina

**Affiliations:** 1 Division of Biology and Genetics, Department of Molecular and Translational Medicine, University of Brescia, Brescia, Italy; 2 Unit of Child and Adolescent Neuropsychiatry, ASST of Garda, Brescia, Italy

**Keywords:** Xq28 trisomy, CNVs, Array-CGH, PAR2, Gilles de la Tourette syndrome, Intellectual disability

## Abstract

**Background:**

Tourette syndrome (TS) is a complex neurodevelopmental disorder (NDD) characterized by multiple chronic involuntary motor and vocal tics with onset during childhood or adolescence. Most TS patients present with additional comorbidities, typically attention deficit hyperactivity disorder (ADHD), obsessive- compulsive disorder (OCD), autism spectrum disorder (ASD) and intellectual disability (ID). Both TS and ID are genetically complex disorders that likely occur as a result of the effects of multiple genes interacting with other environmental factors. In addition to single gene mutations and chromosomal disorders, copy number variations (CNVs) are implicated across many NDDs and ID and contribute to their shared genetic etiology. Screening of CNVs using microarray-based Comparative Genomic Hybridization (aCGH) is now routinely performed in all subjects with NDD and ID.

**Case presentation:**

We report a case of a 12-year-old girl diagnosed with Gilles de la Tourette Syndrome associated to behavior disorders and intellectual disability in particular with regard to language. Array-CGH analysis showed a CNV of a subtelomeric region Xq28 (gain of 260 kb) inherited from the healthy father. The duplication contains two genes, *VAMP7* and *SPRY3* of the PAR2 pseudoautosomal region. FISH analysis revealed that the duplicated segment is located on the short arm of a chromosome 13, resulting in a trisomy of the region. In the proband the expression levels of the genes evaluated in the peripheral blood sample are comparable both those of the mother and to those of female control subjects.

**Conclusions:**

Although the trisomy of the 260 kb region from Xq28 identified in proband is also shared by the healthy father, it is tantalizing to speculate that, together with genetic risk factors inherited from the mother, it may play a role in the development of a form of Tourette syndrome with intellectual disability. This hypothesis is also supported by the fact that both genes present in the duplicated region (*VAMP7* and *SPRY3)* are expressed in the CNS and are implicated in neurotransmission and neurite growth and branching. In addition, similar CNVs have been identified in individuals whose phenotype is associated with autism spectrum disorders or intellectual disability.

## Background

Gilles de la Tourette Syndrome (TS; OMIM #137580) is a childhood-onset neurodevelopmental disorder characterized by the presence of motor and vocal tics. Most patients show improvement at the end of adolescence, but symptoms can persist into adulthood in about a third of cases [1]. The prevalence estimates range from 0.3 to 0.9% [2], with males more frequently affected than females with a ratio of 3:1.

In TS patients co-morbidities are frequent. Among them attention deficit hyperactivity disorder (ADHD), obsessive-compulsive disorder (OCD) [3], learning disorders (LD) [4]. Autism spectrum disorders (ASD), other behavioral and psychosocial problems like anxiety, self-injurious behavior, oppositional defiant and conduct disorders [5] may also be present (reviewed in [4, 6]).

TS as well as ID are genetically complex disorders that likely occur as a result of the effects of multiple genes interacting with other environmental factors. In addition to single gene mutations and chromosomal disorders, copy number variations (CNVs) are implicated across many NDDs and ID and contribute to their shared genetic etiology [7, 8]. Screening of CNVs using aCGH is now routinely performed in all subjects with NDD and ID.

Although previous family studies suggested an autosomal dominant pattern of inheritance, more recent analysis suggest that the mode of inheritance of Tourette Syndrome is more complex. TS heritability is estimated to be 0,77 making it one of the most heritable complex neuropsychiatric disorders [9]. Despite such a strong genetic component, the identification of TS susceptibility genes has proven challenging so far. Although linkage analyses have identified several candidate regions, there is little consensus across studies, suggesting that, as with other neuro-psychiatric disorders, TS is genetically complex and heterogeneous [10].

A limited number of Genome-Wide Association Studies (GWAS) of TS has been published to date either reaching a marginal genome-wide significance or failing to identify genome-wide significant loci for the disease [11]. Mutations involving the *SLITRK1* gene have been identified in a small number of people with Tourette syndrome [12]. *SLITKR1* is highly expressed in the central nervous system and plays a critical part in regulating synapse formation between hippocampal neurons and in differentiation of synapses, helping in neuronal outgrowth. However, most people with Tourette syndrome do not have a mutation in the *SLITRK1* gene.

The impact of rare CNVs on TS disease risk has been assessed in a large sample of > 6500 unrelated individuals of European ancestry. The study demonstrated a global increase in the burden of large, rare CNVs in TS cases compared to controls driven primarily by large, singleton events, in particular large (> 1 Mb) deletions, consistent with marked genetic heterogeneity. The authors also identified two TS susceptibility loci: deletions in *NRXN1* and duplications in *CNTN6* genes, each conferring a substantial increase in disease risk (together are present in 1% of TS cases).

*NRXN1* encodes a presynaptic cell-adhesion molecule involved in synaptogenesis and synaptic transmission at both glutamatergic and GABAergic synapses [13]. Heterozygous mutations of the *NRXN1* gene have been also repeatedly associated with autism and schizophrenia.

Like *NRXN1*, *CNTN6* encodes a cell-adhesion molecule expressed primarily in the CNS that play a role in the formation of axon connections in the developing nervous system [14]. Interestingly, a de novo duplication of the *CNTN6* gene was identified in an autistic patient [15]. As in other complex neuropsychiatric disorders, although there may be a few genes with substantial effects, it is likely that Tourette Syndrome risk involves a combination of common, low-effect and rare, larger-effect variants in multiple genes acting together with environmental factors [16]. Here we report the case of a 12-year-old girl diagnosed with TS associated to ID showing a CNV in Xq28 (duplication of 260 kb) in the subtelomeric region, inherited from the father, that results in a trisomy for the region involved.

## Case presentation

The proband, a 12 and a half years old female, is the only daughter of healthy and non-consanguineous Caucasian parents (the mother is 36 years old, the father 40 at conception). She was delivered by cesarean section for maternal reasons at 39 weeks of a spontaneous and unremarkable pregnancy. Amniocentesis was performed due to advanced maternal age resulting in a normal female karyotype (46,XX). The birth weight was 3000 g (25th percentile), length 50 cm (50th percentile), head circumference 35 cm (50th percentile), Apgar score was 8 and 10 at 1 and 5 min respectively. After birth she was referred as normal: she was breastfed for a few weeks, then artificially, weaned at 6 months. She walked independently at one year, spoke the first words at 18 months. At the admission to the kindergarten, at the age of 3, she was referred to the Child Neuropsychiatry Unit on account of language impairment. Intensive speech therapy was supplied for 2 years, then continued with less frequent sessions during the primary school with slowly but steadily positive results. During the second year of the primary school, at the age of 7, learning disability and behavioral disorders became evident. Cognitive assessment performed by Leiter R scale (specific for nonverbal intelligence) and by WPPSI-III, gave an IQ of 91 and 77, respectively (with VIQ = 68, PIQ =89 at the latter scale). A diagnosis of a neurodevelopmental disorder compatible with Gilles de la Tourette syndrome was made at age of 11 for the presence of motor and vocal tics, hyperactivity, motor and behavioral mannerisms, together with mild intellectual disability. During instrumental work up audiological examination, EEG and brain MRI were performed with normal results. At the last follow-up, when she was 12 years old, physical examination showed a height of 153 cm (35th percentile), weight of 61 kg (91th percentile). She had her first menstruation at the age of 11 years without dysmenorrhea or other related problems. Motor embarrassment and bilateral flat foot, corrected with orthotics, were persistent. The last cognitive assessment performed in 2015 by Leiter R gave an IQ score of 69, with WISC IV scale the results indicated total IQ of 57 (VCI = 60, PRI = 67, WMI = 61, PSI = 85) while the GAI was 59. The proband now attends the second class of the secondary school with a learning support teacher. Pharmacological treatment with Aripiprazole 8 mg/day is ongoing.

## Results

**Fig. 1:**
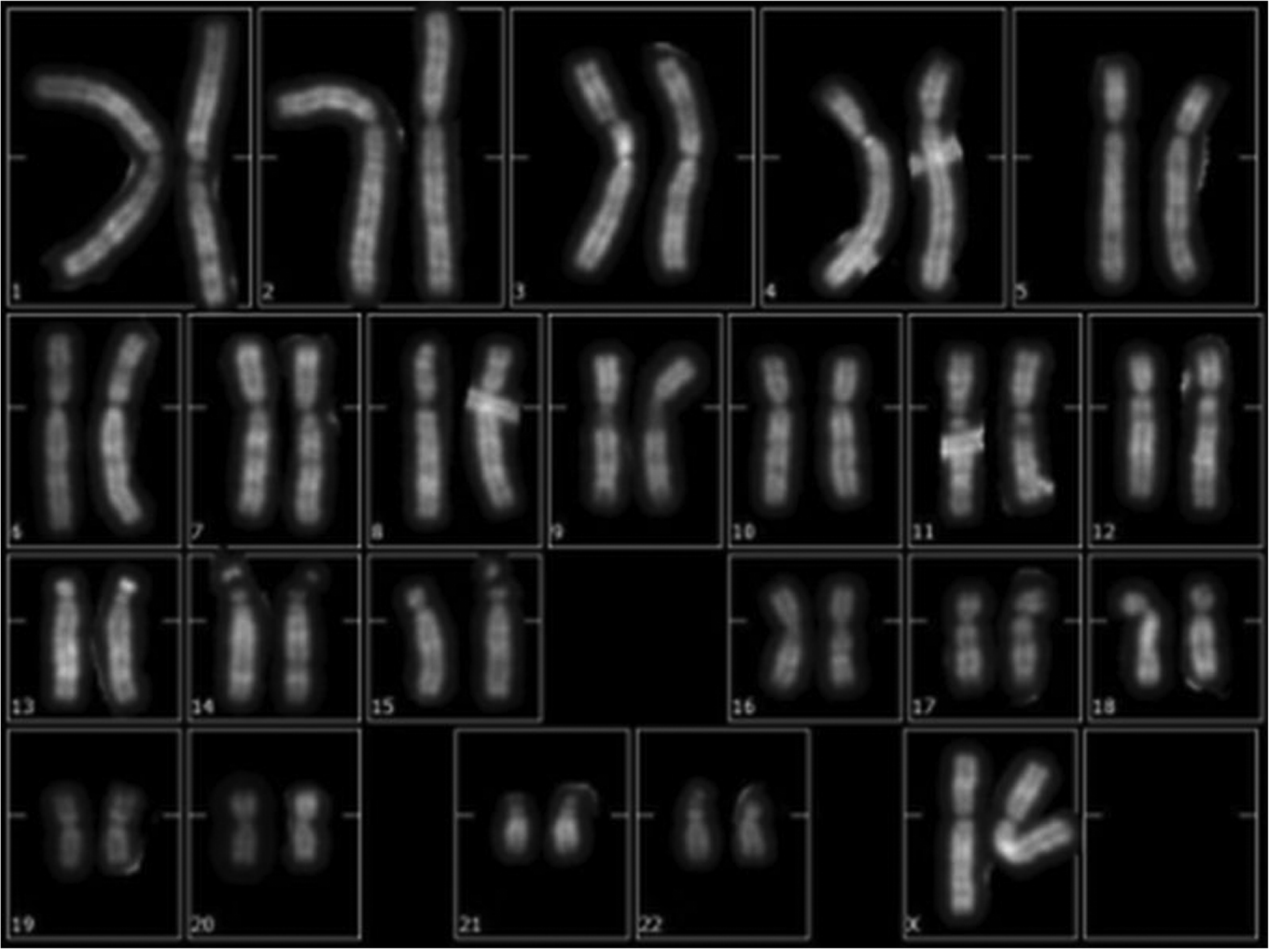
Metaphase of peripheral blood of the proband showing normal chromosome contents with QFQ banding. Cytogenetic analysis was performed using Q-banding at 550 bands resolution, in line with the International System for Human Cytogenetic Nomenclature (ISCN, 2016)

**Fig. 2:**
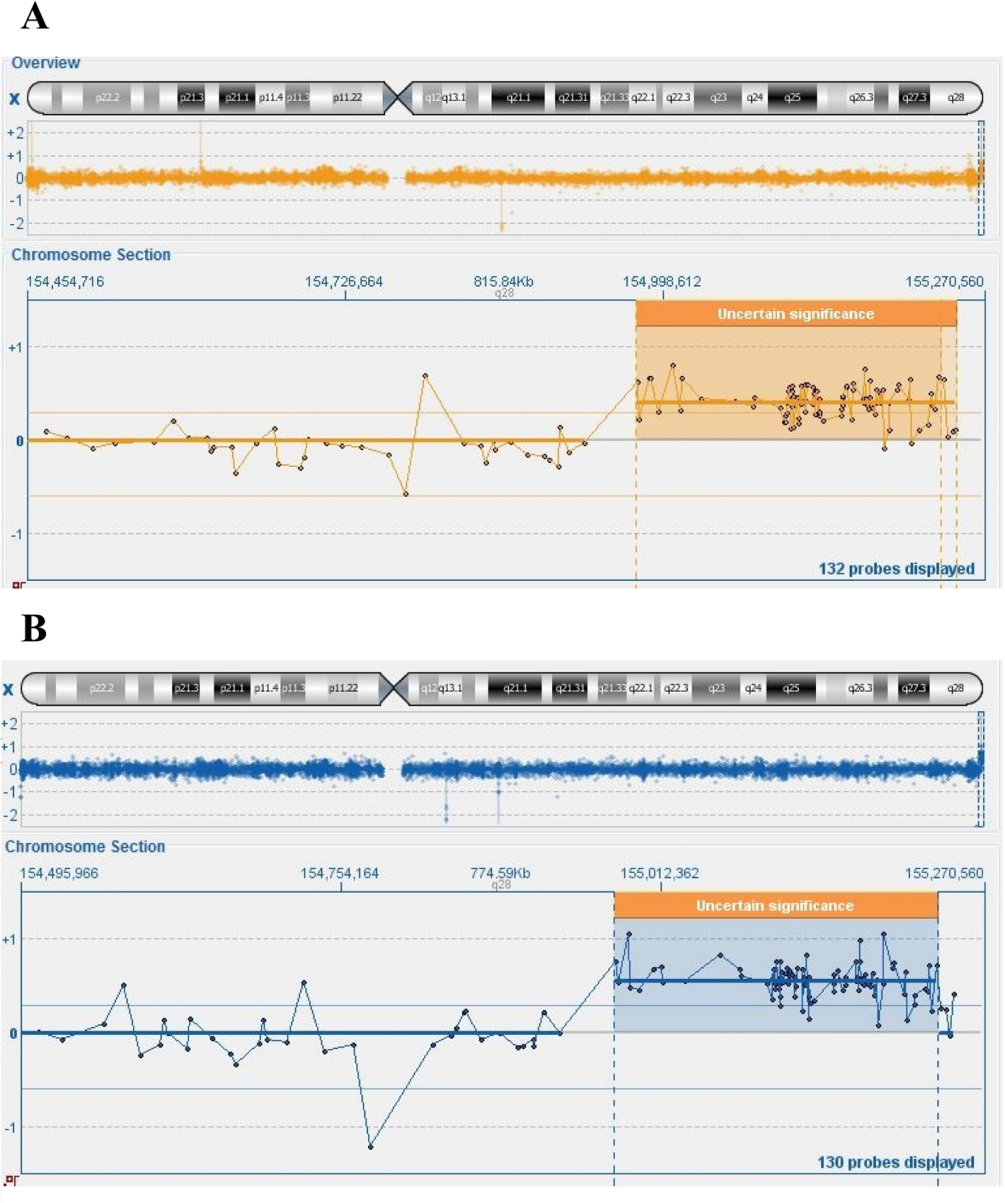
Array-CGH analysis of the proband (**a**) and of the father (**b**) showing a copy number variation of 260 kb of the terminal region of chromosome X. aCGH was performed using the Oxford Gene Technologies CytoSure ISCA v.2 4x180K Microarray Kit (backbone resolution: 1 probe every 25 kb) according to the manifacturer’s recommendations. Promega G152A and 147A were used as female and male reference DNA, respectively. Analysis of aCGH data was performed using the CytoSure Interpret Software (Oxford Gene Technologies). The X chromosome coordinates are relative to the GRCh37/hg19 assembly

Karyotype analysis was performed on peripheral blood lymphocytes of the proband stimulated with phytohaemoagglutinin. The 20 metaphases analyzed by high-resolution QFQ banding (550 bands) revealed normal female karyotype 46,XX (Fig. 1). FRAXA analysis performed by PCR on DNA from peripheral blood revealed that the proband is heterozygous for two *FMR1* alleles with a number of CGG triplets in the normal range (29/30 +/− 1) (data not shown). This result allowed to exclude that the patient is affected by the fragile X Syndrome. Array-CGH analysis, performed using the Oxford Gene Technology 4x180K platform, revealed a copy gain of a 260 kb region in Xq28, at the terminal region of the long arm the X chromosome (Fig. 2A). This region includes the complete sequence of the *SPRY3* and *VAMP7* (also known as *SYBL1*) genes and a portion at the 5′ side of the *IL9R* gene, within the PAR2 pseudoautosomal region. Array-CGH analysis performed on the parents revealed that the terminal Xq28 duplication was inherited from the father (Fig. 2B), while the mother did not present this CNV.

**Fig. 3:**
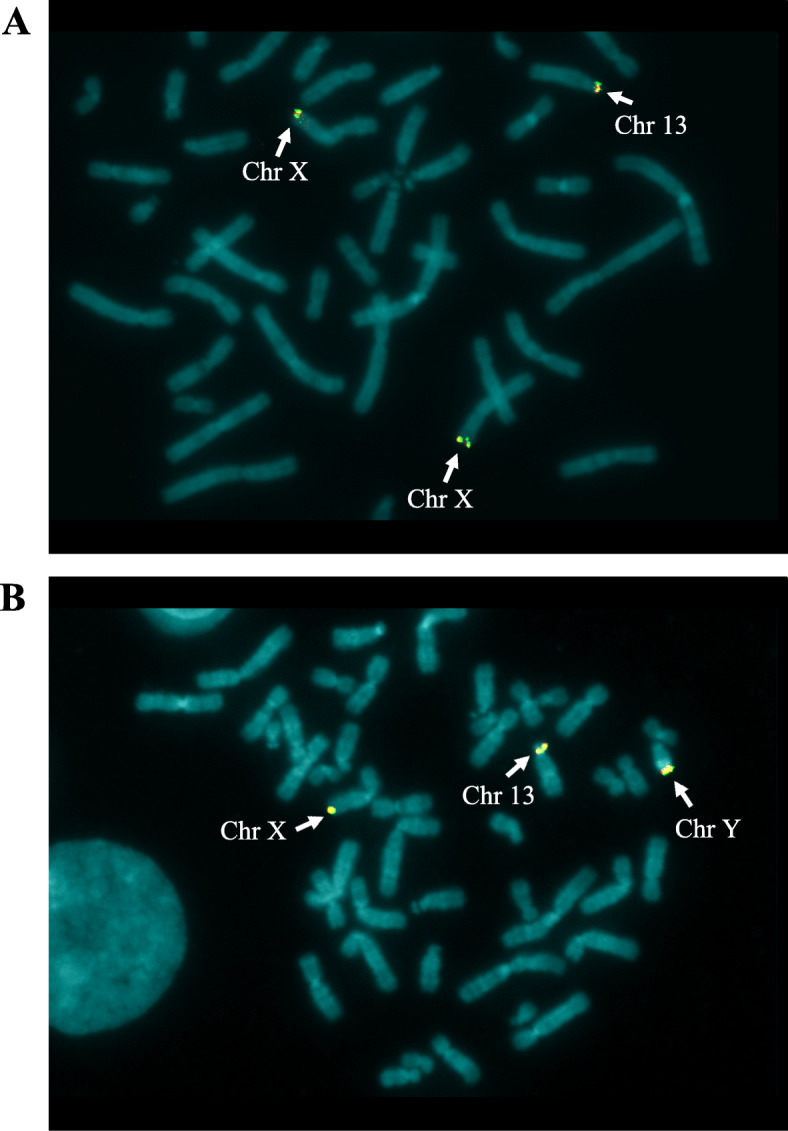
FISH analysis of the proband (**a**) and of the father (**b**) with a probe for the subtelomeric region of chromosome X. FISH analysis was carried out on metaphase chromosomes using the subtelomeric probe (Vysis TelVysion DNA Probes) Xqter (EST Cdy 16c07, GenBank Z43206)

To confirm the duplication and locate its position, FISH was performed on a smear of peripheral blood lymphocytes of the proband using a subtelomeric probe of long arm of chromosome X, which includes the *VAMP7* gene (EST Cdy 16c07, GenBank Z43206). FISH analysis shows the presence of a hybridization signal in the subtelomeric region of both X chromosomes plus an additional one on the short arm of a chromosome 13 in p13, confirming the trisomy of the Xq28 region (Fig. 3A).

**Fig. 4:**
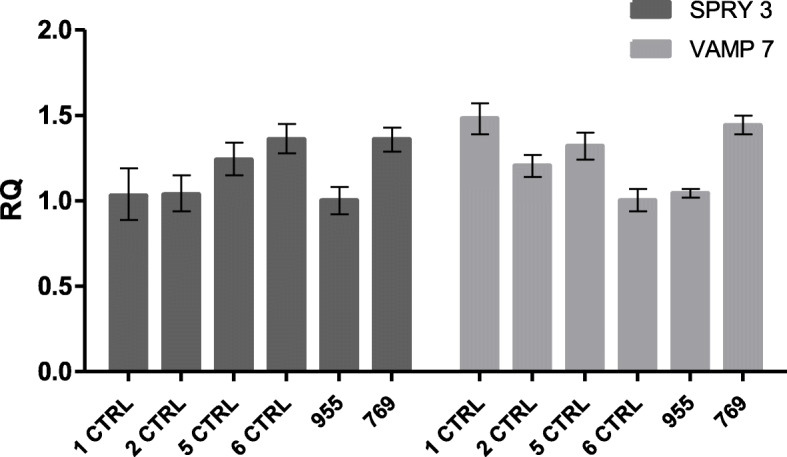
Relative quantification (RQ) of the expression of *SPRY3* (dark grey bars) and *VAMP7* (light grey bars) genes in blood RNA evaluated by qRT-PCR in female control subjects (1–6 CTRL), in the proband (769) and in her mother (955). Unfortunately, the father of the proband was not available to be tested. qRT-PCR was carried out using TaqMan Gene Expression assays for the two genes on RNA from the peripheral blood obtained with the PAXgene Blood RNA Kit (Qiagen)

The final karyotype, expressed in GRCh37/hg19 coordinates, therefore appears to be: 46,XX.arr[GRCh37] Xq28(154974696_155232894)×3.ish der(13)t(X;13)(q28;p13)(EST CdY 16c07 for *VAMP7*+). FISH analysis performed on the father with the same probe used for the proband, shows a result identical to that of the daughter (Fig. 3B). In the specific case the hybridization signals of the Xq subtelomeric probe were present on the X chromosome and on the Y chromosome (being the pseudoautosomal region) and on the short arm of the chromosome 13. This also results in a trisomy of the region of interest. Although it is tantalizing to hypothesize that the neurodevelopmental phenotype of the proband is due to the trisomy of the 260 kb Xq28 region, the same chromosomal imbalance is present in the healthy father. To ascertain if the father is a mosaic for the observed mutation, thus justifying his normal phenotype, a new FISH analysis was performed with the same probe on a larger number of metaphases. All the 100 metaphases analyzed show the presence of the derivative chromosome 13, thus excluding the occurrence of mosaicism, at least in peripheral blood cells. The 260 kb triplicated region is included in pseudoautosomal region PAR2 and contains the entire sequence of *VAMP7* and *SPRY3* genes. qRT-PCR was carried out using TaqMan Gene Expression assays for *VAMP7* and *SPRY3* genes on total RNA from the peripheral blood using the PAX gene Blood RNA kit (Qiagen). The experiment provides no evidence that the expression level of the two genes is altered with respect to that of the mother and of normal female controls (Fig. 4).

## Discussion and conclusions

This study describes the case of a 12 years old girl presenting Tourette Syndrome and intellectual disability in particular with regard to language with a CNV in Xq28 (duplication of 260 kb) in the subtelomeric region, inherited from the healthy father, that results in a trisomy for the region involved. The duplication is fully included in the PAR2 region. The pseudoautosomal regions get their name because any genes within them are inherited like any autosomal genes. PAR1 comprises 2.6 Mb of the short-arm tips of both X and Y chromosomes in humans while PAR2 is at the tips of the long arms, spanning 320 kb. According to the HUGO Gene Nomenclature Committee (HGNC) the PAR2 region contains 4 protein coding genes (*DDX11L16*, *IL9R*, *SPRY3* and *VAMP7*), 5 pseudogenes and 1 gene for a long noncoding anstisense RNA. The two protein coding genes included in the region duplicated in the proband, *SPRY3* and *VAMP7,* undergo X-inactivation and the Y alleles in males are also silent [17, 18].

The *VAMP7* (vesicle-associated membrane protein 7 gene) gene encodes a member of the synaptobrevin family of proteins involved in synaptic vesicle docking, exocytosis, and membrane transport. *Vamp7*-lacking mice have been shown to exhibit increased anxiety, indicating that VAMP7 mediated neurotransmission has an important role in higher brain functions [19]. A reduction of protein levels of VAMP7 selectively inhibit spontaneous neurotransmission both in cultured neurons and in hippocampal slices [20]. Interestingly, a polymorphism in the regulatory region of the gene has been associated with bipolar affective disorders [21, 22].

*SPRY3* is a member of a family of negative regulators of Receptor Tyrosine Kinase (RTK) signaling that is involved in axonal morphogenesis [23]. The gene is highly expressed in central and peripheral nervous system ganglion cells in mouse and human, including cerebellar Purkinje cells and retinal ganglion cells. Transient over-expression or knockdown of *Spry3* in cultured mouse superior cervical ganglion cells inhibits and promotes, respectively, neurite growth and branching [24]. Recent studies suggest that *SPRY3* deregulation may be involved in the pathogenesis of autism [24, 25].

The 260 kb duplication in Xq28 is not reported as polymorphism in the Database of Genomic Variants (DGV), not exceeding 1% [26]. Two cases of copy number gain involving *SPRY3* and *VAMP7* and are reported in the DECIPHER Database [27], which present this CNV as the only variant and whose phenotype is associated with autism or language delay. The duplications have a length of 204 kb and 290 kb, respectively (Table 1). Unlike our case, the probands are male and the inheritance of this CNV is not known. In ClinGen [28], five cases are reported which present this CNV as the only variant and whose phenotype is associated with autism, developmental delay and behavioral anomalies (Table 2). Duplications vary from a minimum of 116 kb to a maximum of 305 kb. The sex of these patients is not known. It is thus conceivable that the genic imbalance in the proband is involved in the pathogenesis of the phenotype. To assess a possible implication of the CNV in the patient’s phenotype, a qRT-PCR analysis was carried out showing that in *whole blood* there is comparable expression of *VAMP7* and *SPRY3* genes between the proband and the mother and the investigated female controls. This is an unexpected finding, considering that the proband carry an extra copy of the two genes on the short arm of the chromosome 13. This result may be due to a position effect, as observed in many cases of unbalanced chromosomal rearrangements [29].These data, however, do not exclude that an altered gene expression may be present in other unexplored tissue, in particular in the CNS.

**Table 1:** Duplications involving the VAMP7, SPRY3 and IL9R genes, highlighted alone or together with other CNVs in the same patient as reported in the Decipher database. The phenotype highlighted in each case, the size of the CNV and its classification/interpretation is also reported

Decipher n.	CNV size	Genes involved	Phenotype	N. of CNV	Classification
283,573	289,76 Kb	*VAMP7-SPRY3-IL9R*	Anomalies of the nervous system	4	Unknown
287,906	272,18 Kb	*VAMP7-SPRY3-IL9R*	Intellectual disability	4	Likely benign
295,447	204,25 Kb	*VAMP7-SPRY3*	Autism	1	Unknown
289,826	224,04 Kb	*VAMP7-SPRY3*	Intellectual disability	4	Likely benign (maternal)
288,136	258,43 Kb	*VAMP7-SPRY3-IL9R*	Intellectual disability	4	Unknown
266,523	258,19 Kb	*VAMP7-SPRY3-IL9R*	Autism	3	Unknown
295,234	232,44 Kb	*VAMP7-SPRY3*	Cognitive impairment	3	Unknown
287,714	147,3 Kb	*VAMP7-IL9R*	?	4	Unknown (de novo)
341,563	289,99 Kb	*VAMP7-SPRY3-IL9R*	Language delay	1	Likely benign
369,986	213,42 Kb	*VAMP7-SPRY3*	Intellectual disability	3	Unknown

**Table 2:** Duplications involving the VAMP7, SPRY3 and IL9R genes, highlighted alone or together with other CNVs in the same patient as reported in the ClinGen database. The phenotype highlighted in each case, the size of the CNV and its classification/interpretation is also reported

NSSV-NSV	CNV size	Genes involved	Phenotype	N° of CNV	Classification
581510–529342	195,2 Kb	*SYBL1-SPRY3*	Autism	1	unknown
581511–532993	305 Kb	*SYBL1-SPRY3-IL9R*	Developmental delay	1	unknown
581512–532924	285,9 Kb	*SYBL1-SPRY3*	Developmental delay	1	unknown
1604719–915617	258,2 Kb	*SYBL1-SPRY3-IL9R* (part.)	Developmental delay	3	likely benign
1601341–915617	258,2 Kb	*SYBL1-SPRY3-IL9R* (part.)	Autism	3	likely benign
1603110–916312	115,6 Kb	*SYBL1* (part.)-*SPRY3* (part.)	Developmental delay	1	likely benign
581513–532995	185,2 Kb	*SYBL1-SPRY3* (part.)	Behavioral anomalies	1	unknown
1609838–931742	131,4 Kb	*SYBL1* (part.)-*IL9R*	Developmental delay	1	benign

The healthy father and the affected daughter present the same rearrangement: where does the phenotypic diversity between the two subjects come from? In the literature are reported several cases of subjects carrying the same chromosomal imbalance but with discordant phenotypes and this is often attributed to incomplete penetrance or variable expressiveness. We can speculate that both the Xq28 CNV and genetic risk factors inherited from the mother are required for the development this form of Tourette syndrome with neurodevelopmental delay.

The accurate follow up of the patient together with additional genomic analysis may provide new insights on the molecular basis of the patient’s phenotype and for genetic counseling.
